# Phylogenomics and Antimicrobial Resistance of Clinical *Bacteroides* Isolates from a Tertiary Hospital in Southern Thailand

**DOI:** 10.3390/antibiotics14111143

**Published:** 2025-11-11

**Authors:** Mingkwan Yingkajorn, Thunchanok Yaikhan, Worawut Duangsi-Ngoen, Chollachai Klaysubun, Thitaporn Dechathai, Sarunyou Chusri, Kamonnut Singkhamanan, Rattanaruji Pomwised, Monwadee Wonglapsuwan, Komwit Surachat

**Affiliations:** 1 Department of Pathology, Faulty of Medicine, Prince of Songkla University, Hat Yai 90110, Songkla, Thailand; 2 Department of Biomedical Sciences and Biomedical Engineering, Faculty of Medicine, Prince of Songkla University, Hat Yai 90110, Songkhla, Thailand; 3 Division of Infectious Diseases, Department of Internal Medicine, Faculty of Medicine, Prince of Songkla University, Hat Yai 90110, Songkhla, Thailand; 4 Division of Biological Science, Faculty of Science, Prince of Songkla University, Hat Yai 90110, Songkhla, Thailand

**Keywords:** *Bacteroides*, antimicrobial resistance, mobile genetic element, phylogenetic analysis

## Abstract

**Background/Objectives**: *Bacteroides* species are key members of the human gut microbiota but can act as opportunistic pathogens. This study investigated the genomic features of clinical *Bacteroides* isolates from southern Thailand. **Methods**: Sixteen isolates were collected from body fluids, tissues, and pus at Songklanagarind Hospital (2022–2024). Whole-genome sequencing was performed on the BGI platform, followed by genome assembly, annotation, average nucleotide identity (ANI), pairwise single-nucleotide polymorphism (SNP) analysis, antimicrobial resistance (AMR) gene profiling, plasmid prediction, virulence screening, and phylogenetic analysis. **Results**: ANI and SNP analysis revealed two clusters: one comprising *B. ovatus*, *B. intestinigallinarum*, and *B. thetaiotaomicron*, and another mainly *B. fragilis* with one *B. hominis* isolate. All isolates were resistant to ampicillin, cephalothin, and penicillin; six *B. fragilis* strains were resistant to all tested antibiotics. The β-lactamase gene *cepA* was detected in all *B. fragilis* isolates, and plasmids were predicted in two genomes. Three virulence types (capsule formation, lipopolysaccharide modification, and stress response) were identified. Phylogenomic analysis confirmed species-level assignments and revealed underrecognized lineages, emphasizing the value of genome-based approaches for accurate classification. **Conclusions**: Clinical *Bacteroides* isolates display diverse resistance and virulence profiles, highlighting the importance of strain-level genomic surveillance.

## 1. Introduction

The genus *Bacteroides* comprises a diverse group of Gram-negative, obligate anaerobic bacteria that are predominant constituents of the human gut microbiota. While generally considered commensals, certain *Bacteroides* species can become opportunistic pathogens, especially in immunocompromised individuals or when the mucosal barrier is ruptured [1]. These bacteria are frequently implicated in a range of clinical infections, including intra-abdominal abscesses, bloodstream infections, and wound infections [2]. Among them, *Bacteroides fragilis* is the most commonly isolated species in clinical practice, recognized not only for its pathogenic potential but also for its remarkable ability to develop resistance to multiple antimicrobial agents [3].

The rise in antimicrobial resistance (AMR) among *Bacteroides* spp. poses a growing clinical challenge, driven by widespread antibiotic use and the ability of these organisms to harbor diverse resistance determinants, including those against aminoglycosides, β-lactams, fluoroquinolones, macrolides, and tetracyclines [4]. The horizontal gene transfer (HGT) of resistance genes via mobile genetic elements (MGEs) such as plasmids, transposons, and insertion sequences further complicates treatment strategies [5]. In addition to AMR, virulence factors such as capsule biosynthesis genes, stress response proteins, and metal transport systems contribute to the pathogenicity of *Bacteroides* and require comprehensive investigation.

Recent advances in whole-genome sequencing (WGS) have enabled high-resolution analysis of bacterial genomes, offering insights into species identification, AMR and virulence gene profiles, phylogenetic relationships, and the presence of mobile genetic elements. Tools such as the Type (Strain) Genome Server (TYGS) and digital DNA–DNA hybridization (dDDH) have improved the accuracy of genome-based taxonomic classification and phylogenomic comparison [6].

In this study, we performed WGS and comparative genomic analysis of 16 clinical *Bacteroides* isolates obtained from patients at Songklanagarind Hospital, Thailand. Our objectives were firstly to determine species-level taxonomy and genomic relatedness using ANI, SNP, and TYGS-based phylogenomics; secondly to identify antimicrobial resistance and virulence gene profiles; and lastly to investigate the presence of plasmids and mobile genetic elements. Although based on a limited number of isolates, this study provides foundational genomic insights into *Bacteroides* species circulating in a tertiary hospital in southern Thailand, serving as a reference for future surveillance efforts.

## 2. Results

### 2.1. Taxonomic Identification and Antimicrobial Susceptibility in Bacteroides spp.

Sixteen isolates of *Bacteroides* spp. were collected from clinical specimens (body fluid, tissue, and pus) collected between 2022 and 2024. Species identification using GTDB revealed nine isolates of *B. fragilis*, four of *B. thetaiotaomicron*, and one each of *B. hominis*, *B. intestinigallinarum*, and *B. ovatus.* All isolates were tested against six antibiotics. Resistance was widespread among the isolates. Six isolates (*B. fragilis* PSU2A, PSU11A, PSU15A, PSU16, PSU18, and PSU20A) exhibited resistance to all tested antibiotics, while the remaining isolates displayed varied resistance patterns. All isolates (100%) were resistant to ampicillin, cephalothin, and penicillin. Tetracycline resistance was observed in 15 isolates (93.75%), with *B. intestinigallinarum* PSU16A being the only susceptible strain. Erythromycin resistance was detected in 14 isolates (87.5%), except for *B. fragilis* PSU12A and *B. thetaiotaomicron* PSU26A. In contrast, chloramphenicol resistance was lower, with only six isolates (37.5%) exhibiting resistance to this antibiotic (Table 1).

### 2.2. ANI and SNP-Based Genomic Relatedness of Bacteroides Isolates

**Figure 1:**
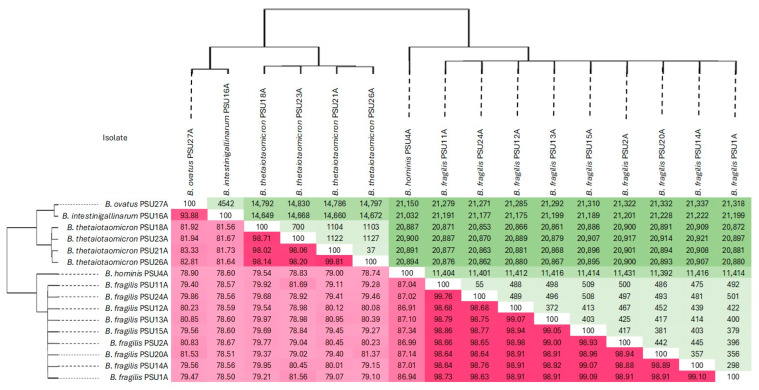
Average nucleotide identity value (red) and number of SNPs from core gene alignment (green) of *Bacteroides* spp.

Average Nucleotide Identity (ANI) values among the 16 *Bacteroides* genomes were calculated using FastANI to confirm species-level taxonomy and assess overall genomic similarity. As shown in Figure 1, pairwise ANI values are visualized using a red color gradient, where darker red indicates higher nucleotide identity (≥95%), consistent with genomes belonging to the same species. Gradual lightening of the red tone reflects decreasing identity between orthologous genomic regions.

In parallel, core-genome SNP distances were calculated and are illustrated using a green color scale, where darker green corresponds to higher SNP counts, indicating greater genetic divergence. The inverse relationship between ANI and SNP distances was evident—genome pairs with high ANI values showed low SNP differences, while those with lower ANI values exhibited more SNP variation, supporting the robustness of both approaches for evaluating genomic relatedness.

Based on these analyses, two major genomic clades were identified. The first clade comprised *B. ovatus*, *B. intestinigallinarum*, and *B. thetaiotaomicron*, whereas the second clade included *B. fragilis* and *B. hominis*, reflecting species-level divergence and phylogenetic grouping consistent across both ANI and SNP metrics.

### 2.3. Antimicrobial Resistance Genes in Bacteroides spp.

**Figure 2:**
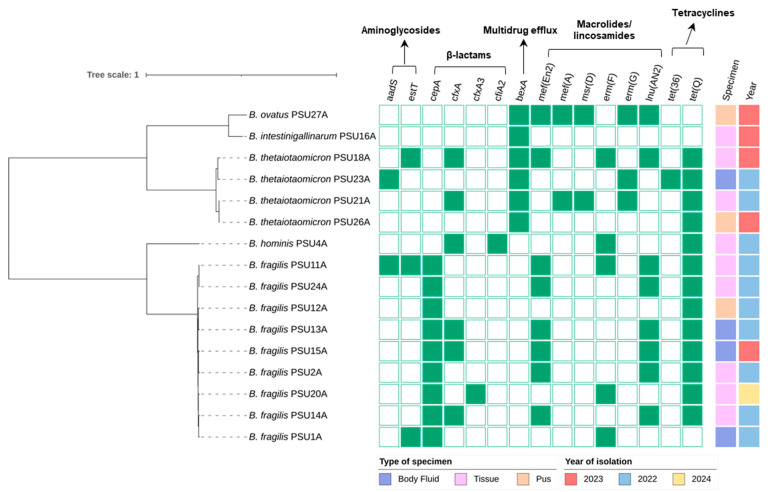
Distribution of antimicrobial resistance (AMR) genes among 16 *Bacteroides* spp. isolates collected from Songklanagarind Hospital, Thailand. The phylogenetic tree was constructed based on core gene alignment (Newick format). The scale bar indicates a branch length corresponding to one nucleotide substitution per site, representing genetic distance among the isolates. Presence and absence of AMR genes are shown in green and white, respectively. Metadata, including specimen type and year of isolation, are displayed on the right. The legend is shown below. Visualization was performed using iTOL.

Among the 16 *Bacteroides* isolates, β-lactamase genes—such as *cepA*, which is consistent with intrinsic resistance to penicillins—were detected in all *B. fragilis* strains. The *cfxA* gene, associated with cephalosporin resistance, was also highly prevalent across various species in the genus, including some isolates of *B. thetaiotaomicron*, *B. hominis*, and *B. fragilis*. Notably, a metallo-β-lactamase gene, *cfiA2*, linked to carbapenem resistance, was identified in *B. hominis* PSU4A, raising clinical concerns. In addition, macrolide resistance genes *erm(F)* and *erm(G)*, along with the tetracycline resistance gene *tet(Q)*, were commonly observed, indicating widespread resistance to multiple key antimicrobial classes. MDR genes were identified among the isolates. In particular, *B. thetaiotaomicron* PSU18A harbored resistance genes across all major antimicrobial classes, while *B. fragilis* PSU11A carried resistance determinants for all classes except multidrug efflux (*bexA*) (Figure 2).

### 2.4. Plasmid and Mobile Genetic Element Detection in Bacteroides spp.

Whole-genome analysis revealed the presence of plasmid and mobile genetic elements (MGEs) in *Bacteroides fragilis* PSU11A and *Bacteroides ovatus* PSU27A. Both isolates were found to harbor a circular plasmid belonging to the *repUS2* family, with an approximate length of 2750 bp (Table 2). In addition, *B. ovatus* PSU27A carried multiple MGEs, including two insertion sequences—*ISBaov1* and *ISOdsp1*—and a composite transposon designated *cn_11070_ISBaov1*. This 11,070 bp transposon harbored three antimicrobial resistance genes: *msr(D)*, *erm(G)*, and *mef(A)*, which are associated with resistance to macrolide–lincosamide–streptogramin (MLS) antibiotics. While *ISBaov1* has been previously reported in *B. ovatus* [7], *ISOdsp1* is identified here for the first time in *Bacteroides* spp.

### 2.5. Virulence Genes in Bacteroides spp.

**Figure 3:**
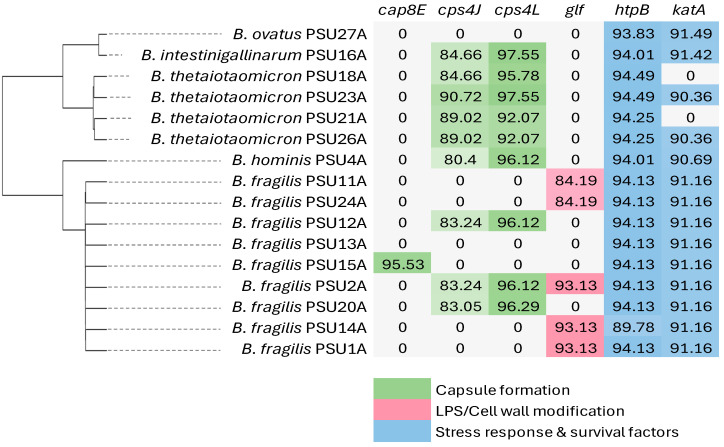
The matrix illustrates the virulence genes identified in 16 *Bacteroides* spp. Different colors represent distinct categories of virulence determinants. The phylogenetic tree on the left was generated based on core gene alignment using Geneious Prime.

Identified six major genes among the 16 *Bacteroides* isolates: *cap8E*, *cps4J*, *cps4L*, *glf*, *htpB*, and *katA* (Figure 3). Genes involved in capsule biosynthesis (*cap8E*, *cps4J*, *cps4L*) were variably detected, with identity values ranging from ~80–97%. The *glf* gene, associated with lipopolysaccharide (LPS) modification, was present in several *B. fragilis* isolates (84–93% identity). Stress response genes were consistently identified: *htpB* (89–94% identity) and *katA* (90–91% identity), highlighting their role in oxidative stress tolerance. Across all isolates, the number of detected virulence genes ranged from 2 to 5 per genome, with *B. fragilis* PSU2A carrying the highest number (five genes).

### 2.6. Comparative Phylogenetic Analysis of Bacteroides Isolates

**Figure 4:**
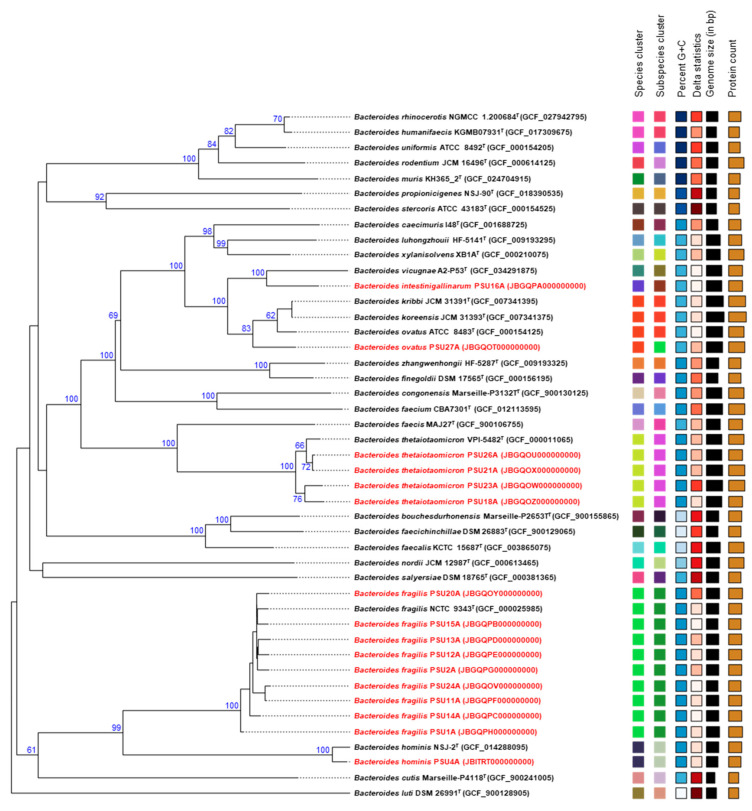
Phylogenetic tree inferred using FastME 2.1.6.1 based on Genome BLAST Distance Phylogeny (GBDP) distances calculated from whole-genome sequences. Branch lengths are scaled according to the GBDP distance formula *d*5. Numbers above branches represent GBDP pseudo-bootstrap support values, based on 100 replicates, with an average branch support of 69.5%. The tree is midpoint-rooted. Genomes of type strains (^T^) were retrieved from the NCBI database, while strains highlighted in red represent isolates obtained in this study.

The genome-based phylogenetic analysis (Figure 4), constructed using FastME 2.1.6.1 with the Genome BLAST Distance Phylogeny (GBDP) algorithm (distance formula *d*5), revealed the genomic relationships among 16 clinical *Bacteroides* isolates obtained in this study and publicly available type strains from the NCBI database. The isolates were distributed into several distinct, species-level clades. Nine clinical isolates clustered closely with the *B. fragilis* NCTC 9343^T^, supported by 100% bootstrap support value, confirming their species-level identity with strong confidence. In contrast, *B. hominis* PSU4A formed a separate lineage, clearly distinct from the *B. fragilis* clade, consistent with its classification as a separate species. Four isolates—PSU18A, PSU21A, PSU23A, and PSU26A—grouped within the *B. thetaiotaomicron* clade, again with strong bootstrap support (100%). Isolate PSU4A clustered with the *B. hominis* type strain and was positioned near the *B. fragilis* clade, with moderate to high bootstrap support (99%). Additionally, PSU27A was grouped with *B. ovatus*, while *B. intestinigallinarum* PSU16A did not have a corresponding type strain available in the database for direct comparison. However, it formed a distinct clade closely related to the *B. vicugnae* A2-P53^T^, with 100% confidence, suggesting a potentially novel or underrepresented lineage within the genus.

## 3. Discussions

This study provides a detailed genomic and phenotypic characterization of 16 *Bacteroides* spp. clinical isolates from Songklanagarind Hospital, Thailand. By integrating antimicrobial susceptibility testing, whole-genome sequencing, and bioinformatic analysis, we examined patterns in antimicrobial resistance, mobile genetic elements, plasmids, virulence genes, and phylogenetic relationships within clinically relevant *Bacteroides* species.

In recent years, *Bacteroides* species have exhibited increasing levels of AMR worldwide. Most strains are now highly resistant to tetracyclines and commonly used β-lactam antibiotics, while moderate resistance has also been reported for moxifloxacin, clindamycin, cefoxitin, and amoxicillin/clavulanic acid [8]. In this cohort, all isolates (100%) were phenotypically resistant to β-lactam antibiotics (ampicillin, cephalothin, and penicillin), which strongly correlated with the presence of *cepA* and *cfxA*. The detection of *cepA* in all *B. fragilis* isolates aligns with its known intrinsic resistance to penicillins, while the widespread occurrence of *cfxA* across *B. thetaiotaomicron*, *B. hominis*, and *B. fragilis* suggests broader dissemination of cephalosporin resistance within the genus. Of particular concern is the identification of the *cfiA2* gene in *B. hominis* PSU4A, a metallo-β-lactamase associated with carbapenem resistance. Although *B. hominis* is less commonly studied than *B. fragilis*, this highlights the potential for an underrecognized reservoir of carbapenem resistance within the *Bacteroides* genus. In global surveillance studies, the *cfiA* gene remains rare even among resistant *B. fragilis* isolates, possibly due to its dependence on upstream insertion sequences for promoter activation [9].

Similarly, phenotypic tetracycline resistance in 15 out of 16 isolates (93.75%) corresponded with the detection of at least one tetracycline resistance gene in nearly all genomes (except for *B. ovatus* PSU27A, *B. intestinigallinarum* PSU16A, and *B. fragilis* PSU1A). These findings also align with the global reports indicating high tetracycline resistance rates (72–92%) among both colorectal and clinical *Bacteroides* isolates, with the *tet(Q)* gene detected in approximately 92% of strains [10].

A moderate occurrence of chloramphenicol resistance (37.5% of isolates) was observed in this study, contrasting with a previous report of 92.8% susceptibility in China [11]. No genes directly associated with chloramphenicol resistance were detected in our isolates, suggesting that the observed resistance may be mediated by nonspecific efflux mechanisms or other undetected resistance determinants. The low rate of chloramphenicol resistance may reflect the limited clinical use of chloramphenicol today, which stems from its well-documented toxicity profile. Given these safety concerns, routine susceptibility testing is uncommon, and contemporary prevalence data remain scarce. Therefore, historical assumptions about chloramphenicol efficacy may no longer be reliable.

Phenotypic results revealed that only two isolates, *B. thetaiotaomicron* PSU26A and *B. fragilis* PSU12A, showed no resistance to macrolide–lincosamide–streptogramin (MLS) antibiotics. Consistently, no genes conferring MLS resistance were detected in these two isolates. The frequent co-occurrence of *erm(F)* and *erm(G)* among the remaining isolates is consistent with MDR genotypes reported in clinical *Bacteroides* from multiple geographic regions [12,13]. These genes are commonly associated with resistance to MLS antibiotics and tetracyclines, reflecting the widespread use of these agents and their role in maintaining MDR within the *Bacteroides* genus [14]. Although a few discordant cases were noted where phenotypic resistance exceeded detected resistance genes, strong genotype–phenotype concordance was observed for β-lactam, tetracycline, and macrolide resistance. This might indicate the clinical reliability of genomic AMR profiling in predicting antimicrobial resistance in *Bacteroides* species. No novel resistance determinants were identified; the presence of MDR gene profiles among Thai *Bacteroides* isolates remains clinically relevant, providing the first genomic characterization of *Bacteroides* isolates from southern Thailand and expanding the limited regional genomic data on anaerobic pathogens.

Beyond resistance gene content, plasmid and mobile element analyses provided insights into possible mechanisms driving resistance dissemination. Two isolates, *B. fragilis* PSU11A and *B. ovatus* PSU27A, carried *repUS2* plasmid replicon markers (~2.75 kb), showing high sequence similarity to the *Bacteroides* plasmid pBI143 (Supplementary Figure S1). Both plasmids encode a *mob* gene, indicating potential for mobilization. In particular, PSU27A harbored several MGEs, including the insertion sequences *ISBaov1* and *ISOdsp1*, as well as a composite transposon (*cn_11070_ISBaov1*) encoding *msr(D), erm(G)*, and *mef(A)*. This composite transposon appears to be novel, as no identical structure has been reported in existing *Bacteroides* genomes. Only single copies of these IS elements were detected in the draft assemblies; however, the precise copy number could not be confirmed due to assembly fragmentation inherent to short-read data. The association of insertion sequences with MLS resistance genes has been documented previously, supporting the role of MGEs in facilitating the mobilization and persistence of antimicrobial resistance determinants in *Bacteroides* [15]. While *ISBaov1* has been previously reported in *B. ovatus* [7], *ISOdsp1* is reported here for the first time in *Bacteroides* spp. Its detection in *Bacteroides* may suggest possible exchange of MGEs among gut microbes, potentially contributing to the spread of resistance-associated genes. As this analysis was based on short-read sequencing, long-read sequencing will be needed to confirm the genomic context and mobility of these elements.

Virulence gene profiling of the 16 clinical *Bacteroides* isolates revealed a conserved yet heterogeneous distribution of genes related to capsule biosynthesis, LPS modification, and oxidative stress responses. Capsule-associated genes (*cap8E, cps4J, cps4L*) were variably detected, associating with capsular polysaccharides in immune evasion and persistence during infection [16]. Interestingly, *cap8E*, originally described in pneumococci, was found in *B. fragilis* PSU15A with 95.53% identity. This may reflect either horizontal gene transfer or detection of partial homologous sequences due to the permissive Abricate parameters, which indicates a limitation of in silico screening and the need for cautious interpretation of in silico virulence gene predictions. The *glf* gene, involved in LPS modification through galactofuranose synthesis, was predominantly detected in *B. fragilis*, where it contributes to immune evasion and abscess formation [17]. This observation indicates that *glf*-mediated LPS modification in *B. fragilis* might represent an adaptive feature, potentially influenced by host-associated or environmental pressures such as inflammation or oxygen availability [18].

Stress response genes, particularly *htpB* and *katA*, were consistently identified across isolates. *htpB*, previously reported as a heat shock protein in *Legionella pneumophila* [19], was present in all *Bacteroides* genomes in this study, reflecting a potential role in protein stabilization under stress. Additionally, *katA*, encoding catalase A, was detected in most of the genomes. Although previous studies report its regulation by the peroxide stress regulator PerR, which responds to intracellular metal balance and oxidative conditions [20], the *perR* gene was not identified in our draft genomes. Notably, the *bft* (*Bacteroides fragilis* toxin) gene was not detected in any of the isolates, suggesting the absence of enterotoxigenic *B. fragilis* (ETBF) lineages in this collection. Despite the small cohort, our study highlights the multifactorial nature of *Bacteroides* pathogenicity, driven by virulence and antimicrobial resistance determinants. While many strains remain benign commensals, others harbor traits that may confer pathogenic potential, emphasizing the importance of strain-level genomic surveillance. In silico approaches provide useful preliminary insights, but larger datasets with experimental validation are needed to confirm gene expression and functional roles.

ANI- and SNP-based analyses delineated two principal clades: one comprising *B. fragilis* and *B. hominis*, and another containing *B. thetaiotaomicron*, *B. ovatus*, and *B. intestinigallinarum*, consistent with established species-level genomic distinctions *(*Figure 1*).* The concordance between ANI and SNP clustering points out the strength of combining whole-genome identity with fine-scale variation for robust taxonomic and evolutionary analysis [21]. To complement this, genome-based phylogenetic reconstruction using the GBDP approach further confirmed these groupings and clarified deeper taxonomic relationships within the genus *Bacteroides*. While ANI and SNP analyses are ideal for confirming species-level identity and intra-species diversity, GBDP provides a broader phylogenetic perspective, enabling validation and resolution of interspecies relationships.

Genome-based phylogenetic analysis revealed that the 16 *Bacteroides* isolates from Songklanagarind Hospital span multiple distinct taxonomic groups (Figure 4). Nine isolates clustered tightly with the *Bacteroides fragilis* type strain, with 100% bootstrap support values, confirming their classification as *B. fragilis*. Interestingly, isolate PSU4A clustered with the *Bacteroides hominis* type strain and was designated as *B. hominis*, although it shared 99% bootstrap support with the *B. fragilis* clade. *B. hominis* is genomically related to *B. fragilis*; however, recent genome-based studies have demonstrated that Division II strains (now recognized as *B. hominis*) are phylogenetically distinct from Division I strains (true *B. fragilis*) based on ANI and accessory gene content [22]. This clear separation emphasizes the accuracy of current taxonomic classification and highlights *B. hominis* as a potentially underrecognized species in clinical infections.

Four isolates (PSU18A, PSU21A, PSU23A, and PSU26A) grouped with the *B. thetaiotaomicron VPI-5482* type strain, also with bootstrap support of 100%. *B. thetaiotaomicron* is well known as a dominant member of the human gut microbiota, where it plays an important role in maintaining intestinal homeostasis and modulating interactions with other microbes [23]. Meanwhile, isolate PSU27A was confidently classified as *B. ovatus,* while *B. intestinigallinarum* PSU16A did not have a corresponding type strain in the reference database. However, its close phylogenetic relationship to *B. vicugnae* A2-P53^T^ suggests a potentially underreported or emerging lineage within the genus *Bacteroides* [24]. Despite providing valuable genomic insights, the small sample size limits statistical and epidemiological generalization. The high-resolution genomic data serve as an essential baseline for subsequent large-scale investigations and comparative analyses of Bacteroides in Thailand and other regions. Expanding future sampling will enable robust association studies between genotype, phenotype, and clinical outcomes.

## 4. Materials and Methods

### 4.1. Isolates Collection, Antimicrobial Susceptibility Testing, and DNA Extraction

Sixteen *Bacteroides* isolates were collected from patient specimens at the Bacteriology and Mycology Unit of Songklanakarind Hospital between 2022 and 2024. The isolates were obtained from various clinical specimen types, including four from body fluid, nine from tissues, and three from pus. Clinical specimens were initially inoculated into thioglycolate broth and incubated at 35 ± 2 °C. Broths showing microbial growth were subcultured onto Anaerobic Blood agar plates and subsequently incubated at 35 ± 2 °C for 48 h under anaerobic conditions using the AnaeroGen™ gas-generating system (Oxoid, Basingstoke, UK) in anaerobic jars. After incubation, colonies displaying typical *Bacteroides* morphology were selected for further identification.

Isolate identification was performed using matrix-assisted laser desorption ionization time-of-flight mass spectrometry (MALDI-TOF MS) analysis (Bruker, Billerica, MA, USA). Identification results were interpreted using the manufacturer’s database (MBT Compass 4.1). Genomic DNA was extracted from confirmed *Bacteroides* isolates using the AnaeroPack-Anaero (Mitsubishi Gas Chemical Co., Inc., Tokyo, Japan). Extracted DNA was stored at −20 °C until further analysis. DNA concentration and purity were assessed using a Qubit^TM^ Fluorometer (Thermo Fisher Scientific, Waltham, MA, USA) prior to sequencing.

Antimicrobial susceptibility of anaerobic bacteria was assessed using the Modified Broth-Disk Method as described by Wilkins and Thiel [25]. Brain Heart Infusion (BHI) broth was used as the culture medium. The antibiotic disks tested included penicillin (10 units), ampicillin (10 μg), cephalothin (30 μg), tetracycline (30 μg), chloramphenicol (30 μg), and erythromycin (15 μg). A positive control consisted of the test organism inoculated in BHI broth without any antibiotic, while a negative control comprised sterile BHI broth with no bacterial inoculation and no antibiotic. Multidrug resistance (MDR) was defined as resistance to three or more different classes of antibiotics among the six tested. Isolates not meeting this criterion were classified as non-MDR.

### 4.2. Bacteroides Genome Sequencing

The genomic DNA extracted from *Bacteroides* isolates was sequenced using BGI sequencing technology. High-quality DNA samples were quantified using a Qubit fluorometer (Thermo Fisher Scientific) and checked for integrity using agarose gel electrophoresis. Approximately 1 µg of DNA was used to construct sequencing libraries with the MGIEasy FS PCR-Free DNA Library Prep Set (MGI Tech Co., Ltd., Shenzhen, China), following the manufacturer’s protocol. The library preparation involved DNA fragmentation, end repair, adapter ligation, and size selection to produce libraries with an average insert size of 350 bp. Libraries were then sequenced on the BGISEQ-500 platform (MGI Tech Co., Ltd.) with 100 bp paired-end reads.

### 4.3. Genomic Characterization Using Bioinformatics

The genomes of *Bacteroides* spp. isolates were analyzed using the Bactopia pipeline (v1.4.0) [26]. Raw sequencing reads in FASTQ format underwent quality control using FastQC v0.12.1 [27], and low-quality reads and adapter sequences were trimmed using Fastp v0.23.4 [28]. High-quality reads were assembled into draft genomes using Shovill v1.1.0 [29]. The assembly results can be assessed in Supplementary Table S1. Antimicrobial resistance (AMR) genes were identified using AMRFinderPlus (v.3.6.7) against the NCBI reference database [30]. Species-level similarity was assessed by calculating average nucleotide identity (ANI) using FastANI v1.34 [31], while single-nucleotide polymorphism (SNP) distances were estimated using snp-dists v0.7.0 to distinguish closely related strains [32]. Genome annotation was performed using Prokka v1.14.6 [33], and taxonomic assignment was carried out with the Genome Taxonomy Database Toolkit (GTDB-Tk v.3.0) [34]. Phylogenetic trees were generated in Geneious Prime v2023.1.2 [35] based on Newick-formatted files and visualized using the Interactive Tree of Life (iTOL) v7 [36]. Virulence-associated genes were screened using Abricate v1.0.1 against the Virulence Factor Database (VFDB). An initial permissive nucleotide identity threshold of 20% was applied to capture distant homologs and reduce false negatives during the preliminary search, acknowledging that virulence-associated genes may exhibit high sequence divergence across *Bacteroides* species. However, only hits with >80% nucleotide identity were retained and interpreted as true virulence determinants to ensure specificity and biological relevance [37].

### 4.4. Plasmid and Mobile Genetic Element Detection

PlasmidFinder was used with default parameters to screen for plasmid replicons in the assembled genomes. Candidate plasmid sequences were further validated through BLASTn against the NCBI nucleotide database, applying filtering criteria of ≥80% query coverage, ≥80% identity, and *E-value* ≤ 1 × 10^−5^. To investigate mobile genetic elements (MGEs), the MobileElementFinder tool (v1.0.3) provided by the Center for Genomic Epidemiology (https://cge.food.dtu.dk/services/MobileElementFinder/) [38] was used on genomes of plasmid-carrying isolates, specifically *Bacteroides fragilis* PSU11A and *Bacteroides ovatus* PSU27A.

### 4.5. Phylogenetic Analysis

Phylogenetic analysis was performed using FastTree (v.1.0) [39] based on core gene alignments derived from Prokka-annotated genome files (.gff). A maximum-likelihood tree was constructed and visualized using Geneious Prime. Additionally, whole-genome-based taxonomic classification was performed using the Type (Strain) Genome Server (TYGS; https://tygs.dsmz.de), a freely available platform for microbial phylogenomics [6]. The analysis included all 16 *Bacteroides* isolates in this study, along with 28 type strain genomes from representative *Bacteroides* species. Intergenomic distances were computed and used to construct a balanced minimum evolution tree via FASTME (v2.1.6.1), with branch support values calculated from 100 pseudo-bootstrap replicates.

## 5. Conclusions

This study provides comprehensive genomic and phenotypic insights into 16 *Bacteroides* clinical isolates from Thailand, revealing high levels of antimicrobial resistance and diverse taxonomic identities. The integration of AST, WGS, and comparative genomics uncovered resistance genes, virulence-associated factors, and mobile genetic elements contributing to both antimicrobial resistance and potential pathogenicity. Phylogenomic analyses using ANI, SNP, and GBDP approaches enabled precise species-level classification and revealed underrecognized or emerging taxa such as *B. hominis* and *B. intestinigallinarum*. These findings emphasize the value of genome-based analysis for accurate species classification and resistance gene detection. Although limited in sample size, this study establishes a genomic reference framework for *Bacteroides* isolates in Thailand and supports the development of future large-scale surveillance studies.

## Figures and Tables

**Table 1:** Antimicrobial susceptibility test in *Bacteroides* spp. in this study.

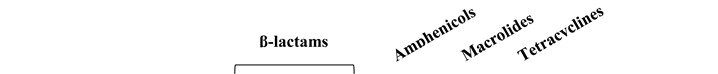							
Isolates	AMP	CH	PEN	CHL	ERY	TET	Resistance Category
*B. fragilis* PSU1A	R	R	R	S	R	R	MDR
*B. fragilis* PSU2A	R	R	R	R	R	R	MDR
*B. hominis* PSU4A	R	R	R	S	R	R	MDR
*B. fragilis* PSU11A	R	R	R	R	R	R	MDR
*B. fragilis* PSU12A	R	R	R	S	S	R	Non-MDR
*B. fragilis* PSU13A	R	R	R	S	R	R	MDR
*B. fragilis* PSU14A	R	R	R	S	R	R	MDR
*B. fragilis* PSU15A	R	R	R	R	R	R	MDR
*B. intestinigallinarum* PSU16A	R	R	R	R	R	S	MDR
*B. thetaiotaomicron* PSU18A	R	R	R	R	R	R	MDR
*B. fragilis* PSU20A	R	R	R	R	R	R	MDR
*B. thetaiotaomicron* PSU21A	R	R	R	S	R	R	MDR
*B. thetaiotaomicron* PSU23A	R	R	R	S	R	R	MDR
*B. fragilis* PSU24A	R	R	R	S	R	R	MDR
*B. thetaiotaomicron* PSU26A	R	R	R	S	S	R	Non-MDR
*B. ovatus* PSU27A	R	R	R	S	R	R	MDR
Resistance (%)	100	100	100	37.5	87.5	93.75	

Abbreviations: AMP; ampicillin, CH; cephalothin, CHL; chloramphenicol, ERY; erythromycin, PEN; penicillin, TET; tetracycline, MDR; multidrug resistance.

**Table 2:** Detected plasmids and mobile genetic elements (MGEs) in *Bacteroides* spp.

Isolate	Name	Type	ARGs	Length (bp)
*B. fragilis* PSU11A	*repUS2*	Plasmid	-	2751
*B. ovatus* PSU27A	*repUS2*	Plasmid	-	2752
	*ISBaov1*	Insertion sequence	-	1594
	*ISOdsp1*	Insertion sequence	-	1098
	*cn_11070_ISBaov1*	Composite transposon	*msr(D)*, *erm(G)*, *mef(A)*	11,070

## Data Availability

The assembled genomes of all *Bacteroides* genome sequencing in this study have been deposited in the NCBI GenBank under BioProject number PRJNA1150746, with BioSample numbers SAMN43295773 to SAMN43295788.

## Supplementary Materials

The following supporting information can be downloaded at https://www.mdpi.com/article/10.3390/antibiotics14111143/s1, Supplementary Table S1. Assembly parameter of 16 *Bacteroides* spp. genomes. Supplementary Figure S1. Sequence similarity analysis of plasmid pBI143 from *Bacteroides fragilis* strain IB143 and plasmid repUS2 from *B. fragilis* PSU11A and *B. ovatus* PSU27A. Annotation was performed using Prokka, and the annotated sequences were visualized using the Clinker tool. The identity percentage between sequences is represented by a grayscale gradient, with black indicating 100% identity. Mobilization (mob) genes are highlighted in purple.
